# Memoirs of Military Surgery and Campaigns

**Published:** 1821-09-01

**Authors:** 


### Medico-Chirurgical Review,

AND

JOURNAL OF MEDICAL SCIENCE.

(Analytical Series.)

Nec tibi quid liceat sed quid fecisse decebit

Occurrat mentemque domat respectus honesti. CLAUD.

VOL. II.] SEPTEMBER 1, 1821. [No. 6.

### I.

### BARON LARREY'S RUSSIAN CAMPAIGN.

*Memoires de Chirurgie Militaire, et Campagnes du Baron D. J. Larrey, Chirurgien en Chef, &c.* i. e. *Memoirs of Military Surgery and Campaigns.* By BARON LARREY, Surgeon in Chief, &c. &c. &c. Vol. IV. pp. 500, with numerous plates. Paris, 1817.

" Eo adductus sum ut multis meorum æqualium hinc indè errantibus viam monstiarem et aliquantulum munirem." *Baglivi.*

THE Gallo-Russian Campaign of 1812 is the most memorable event, political, moral, or military, of the age in which we live. The Roman Empire fell by a series of declensions, occupying many centuries; and consequently its domination faded away by almost imperceptible degrees. But the colossal power of France burst by its own plethoric strength, and its tottering fragments crumbled into dust in the short space of a few months, overwhelmed by famine and fire;—by the elements of Nature, and the sword of the enemy! During this desperate and disastrous expedition, there were many indications (as will presently be shown) of the finger of Providence, which left prophetic impressions on some of the most *philosophic* hearts accompanying the enterprize; and the moral lesson which it has bequeathed to the world will probably operate and be quoted, till the latest posterity. In a medical point of view, the Russian campaign is extremely interesting, and the valuable experience which it yielded to surgery in particular, has, as yet, been withheld from the great body of the British profession.

As it is highly improbable that the volume before us shall ever be translated into our language, we shall possibly be excused for dedicating a moderate space to its analysis, even if it should somewhat exceed that usually allotted to works of equal size, published in this country.

Baron Larrey is an interesting writer, and often digresses, from strictly professional subjects, to paint the eventful scenes around him. He must, indeed, be more or less than man, who could act the stoic on such occasions, or pass unnoticed the tremendous conflicts, and all the multiplied horrors of war, with its attendant train of "plague, pestilence, and famine."

Sunt lachrymæ rerum et mentem mortalia tangunt!

Although we have read with intense interest the Baron's detail of medical and military operations and movements during the advance and retreat of the French army, yet we dare not indulge in more than a most rapid sketch of this portion of the volume, which occupies a space of nearly two hundred pages. We are disposed to hope that few of our readers will wish that the sketch which we are about to lay before them had been omitted, or much curtailed.

Such an effective army as perhaps the world never before produced, consisting of 400,000 men, infantry and cavalry, with Napoleon and nearly all his generals at their head, presented themselves on the banks of the Vistula, in May 1812. This torrent of warriors proceeded from the Vistula to the Niemen, without interruption, crossing the latter river, and directing their march from thence to Wilna, the capital of Lithuania. On this latter route they first began to suffer, from the badness of the roads, and the liquor of the country, which last proved fatal to great numbers of the young conscripts who indulged immoderately in its use. The French entered Wilna on the same day that Alexander left it, after some trifling skirmishes, which produced 150 wounded. Among these there were a few, which the Baron thought worthy of detail.

"A Polish officer, who had been wounded about 24 hours, was brought to Baron Larrey, his whole body swelled, to an enormous size, by general Emphysema. The skin was so distended that his limbs were completely inflexible, and all appearance of the joints effaced. The eyes were covered, and the lips so tumefied that nothing could be got into the mouth. The pulse and respiration were scarce perceptible—the anxiety extreme, the voice feeble and interrupted—in short, this officer appeared to be in the most imminent peril. A cossack's lance had entered obliquely under the inferior angle of the left scapula, and wounded the lungs. Although the external and internal orifices of the wound were far from being parallel, the surgeon had applied adhesive plasters, and closed the external opening. The air constantly escaping from the lungs, had therefore distended the whole cellular tissue of the body. The Baron tore off the dressings, cleared the wound of all transverse filaments or frænula (brides) and thus rendered the openings in the pleura and integuments parallel.* Cupping glasses were applied over the wound, which were quickly filled with air and blood. The lips of the wound were then brought together, and maintained by proper bandages. Cupping glasses and scarificators were next applied successively over all parts of the body to which they could have access; and where they could not, incisions were made with the knife. By this treatment the officer was snatched from the jaws of death, and ultimately recovered perfectly."

Before marching from Wilna, Napoleon ordered a grand review of his army, at which Baron Larrey was present, with all his medical staff and ambulances. The air was hot and calm—"le temps était chaut et calm;" yet at the instant the trumpet announced the approach of the Emperor, the thunder roared, and such a horrible tempest of rain and hail burst from the skies as unhorsed the cavalry soldiers, dispersed the horses, and actually drove the army off the field! "Lorsque la trompette donna le signal de l'arrivée du chef, des coups de tonnere se firent entendre; une effroyable tempete se déchaina sur la terre; le ciel était obscurée au point qu'on ne se reconnaissait à une courte distance, qu'à la lueur des eclairs. Une forte grêle lancée avec violence par les vent impetueux, fit rompre les signes, &c. Les chevaux affrayés cherchaient à s'enfuir, et se jetaient les uns sur les autres, &c." Baron Larrey never before witnessed such a tempest, and all things considered, there was no great superstition evinced by the following question:—"Etait-ce le sinistre présage des malheurs qui nous attendaient?" It would have been wise for Napoleon, though unfortunate for Europe, had he viewed this commotion of the elements as a warning to rein his ambition.

At Witepsk there was some sharp fighting between the French and Russian armies, and 45 amputations were performed. All those who were operated on within the first 24 hours, were successful. Of those, on the other hand, who were amputated on the 3d, 4th, or 5th day, many died.—

* The French term for clearing a wound in the above manner is "*debridement*," which we cannot otherwise translate than by the aukward phrase "*unbridling*" a wound. For conciseness, however, we shall probably, in this article, use the word "debride,' or "debridle," hereafter, to signify the above process.—*Rev.*

This circumstance, the Baron observes, has long been familiar to military surgeons—" et il ne peut rester aucune doute sur la nécessité de faire l'amputation sur le-champ lorsqu'elle est indiquée." 25. Among the remarkable wounds in this action was that of an officer who had a musket ball lodged in the bladder. Lithotomy (if the term be allowed) was performed, and the patient was well before the 30th day. The Baron amputated a Russian soldier at the hip-joint. On the 25th day the wound was nearly healed. But the total failure of provisions at this time brought on fever and dysentery in this and many other wounded men, of which disease the unfortunate Muscovite died on the 30th day after the operation.

The French army pursued the Russian, with rapid strides, to the banks of the Nieper, where the latter made a determined stand, within the fortifications of Smolensko, a very strong town, situated on a bold promontory, washed by the abovementioned river. Thirty thousand Russians occupied Smolensko and the neighbouring heights. The French carried all the positions, one after another, at the point of the bayonet, after 24 hours of the most sanguinary conflict our author ever witnessed. The gates, the breaches, and the principal streets of Smolensko were filled with dead, and dying—principally Russians! The French had twelve hundred killed, and six thousand wounded. The loss of the Russians was incalculable!

It is melancholy to think that even before *this* battle, which may be considered as the *first* of the Russian campaign, the French army was almost completely destitute of every kind of dressings, &c. for their wounded men! "Ici, comme a Witepsk, nous fumes en penurie de toutes sortes de secours materiels pour les pansemens de blessées." For lint they were obliged to substitute paper—and the parchment archives of the city were converted into splints. The cold-blooded cruelty of Napoleon, in thus making no provision for those wounded warriors who bled to satiate his ambition, we cannot find proper terms to execrate! For this and other crimes, eternal justice has wisely spared his life, that the vulture of revenge may long and deeply prey on his vitals beneath the burning rays of a tropical sun. May he live a thousand years, as a moral lesson to ambition and tyranny!

What the flames spared of this ill-fated city, was destroyed by plunder—" devenue la proie des flammes ou du pillage." There was, of course, every variety of wounds. One of the most remarkable cases was that of a corporal in the 13th Regiment of the line. A bullet of large calibre shattered to pieces the head of the left humerus, the clavicle, and the scapula, the bony fragments of which parts were reversed on the back, together with the torn and mashed soft parts:—"les fragmens osseux étaient renverses sur le dos, avec les parties molles décherées et attrites." This wound presented a most frightful aspect, and the poor soldier begged hard that the shattered parts, together with numerous splinters sticking in the flesh, might be cut away. Baron Larrey tied the axillary artery, and then detached bones and lacerated muscles, skin, &c. though with little hope of success, bringing the edges of such a tremendous wound as nearly together as he could with adhesive straps and bandages. On the 35th day after the operation, when Smolensko was evacuated, the patient was in rapid progress to recovery. But whether he perished afterwards in the dreadful retreat, is unknown. During the first 24 hours, Baron Larrey performed eleven amputations *at the shoulder joint alone*, nine of which perfectly recovered—the other two died of dysentery. We must pass over the numerous other operations which our experienced author performed.

No sooner had he crossed the last branch of the Nieper, than Baron L. was seized with a kind of sea-sickness, which tormented him for months afterwards, and was accompanied with some painful optical illusions, as an appearance of tremulous motions in the horizon and earth around him, for which he could no otherwise account than by the perpetual sight of huge masses of men in constant motion around him on the immense plains of Russia. From the capture of Smolensko, the French found every town and village in flames as they advanced. "Ici commencerent pour nous les privations de toute espece. Cet exemple sévère devait nous avertir des maux qui nous attendaient sur le reste de la route." But no—drawn along by an irresistible power, (entrainés par une puissance invincible,) Napoleon rushed towards his fate, and dragged, alas! 300,000 men to theirs!

They reached Viasma, a considerable depôt for the two Russias, but there all the immense magazines of oil, brandy, coffee, sugar, furs, &c. were blazing to the skies. It was with difficulty the army could pass through the conflagrating ruins. The town of Giad was in the same condition, and here they learnt that the whole Russian army was strongly intrenched at Mozaisk, and there determined to give final battle. Orders were therefore issued to prepare for a fiercer conflict than they had yet attempted, to the utter consternation of Baron Larrey, who had neither surgeons nor surgical materials for this new scene of carnage. Twenty-four hours' sojourn at Giad, however, gave our author some time to prepare, and no human being, we verily believe, ever exerted more courage, patience, and humanity, on these trying occasions, than did our worthy and able Baron. Thirty-six hours' march brought the French army before the position of the enemy, and the 7th September was pitched on for deciding the fate of the campaign. At break of day more than two thousand pieces of artillery opened, on both sides, and commenced their destructive work. The battle lasted with indescribable fury from six in the morning till nine in the evening; and when, as night approached, both armies made one desperate and last effort, the balance of fate vibrated for some minutes, as though uncertain which way to incline! "La victoire resta même quelques momens incertaine." Napoleon's *evil* star prevailed—and his destiny was sealed by the overthrow of the Russians. We say *evil* star—for had the Russians defeated him, he might have fallen back on his resources, preserved the greater part of his army, and still wielded the sceptre.

On this bloody day the French had upwards of forty generals, and from twelve to thirteen thousand men, placed *hors de combat*, even by their own computations! That of the Russians was supposed to be upwards of twenty thousand! The difficulties and fatigues which the medical officers experienced, on this occasion, were indescribable; and the sudden change to cold and tempestuous weather added to their other embarrassments. Of eleven shoulder-joint amputations, two only died. The most remarkable case in this class, was that of a field officer, who mounted his horse the moment after the operation, and continued his march the whole way back to France without ever halting a day. He perfectly recovered.

In many cases, during this battle, Baron Larrey found the thigh shattered too high up to perform the amputation with the circular incision, and yet not so high as to require extirpatian at the hip-joint. He therefore made the flap operation on a level with the great trochanter, or very near that apophysis. Here the Baron performed the hip-operation on a subaltern officer with success; but the patient was lost in the retreat afterwards. In this place the Baron describes a most dangerous accident which often wears an aspect, at the beginning, by no means threatening. It is when a ball strikes the femur a little above the knee-pan, and either passes through the limb, with fracture of the bone of course, or lodges in the ham. In such cases he has generally found the condyles of the femur separated, and consequently nothing but amputation affords any chance of life. We shall abridge a case in elucidation.

Count Sackoveninsk, a Russian colonel (a prisoner) was carried, with several of his countrymen, to the General Ambulance. He had received a musket bullet just above the left knee, which fractured the femur and lodged under the integuments of the ham. M. Bancel, after extracting the ball, was about to dress the wound and apply the fracture apparatus; but Larrey was first requested to see the Colonel. On a slight view there appeared nothing particular or very dangerous in the case; but on more accurate investigation the Baron had reason to believe that a perpendicular fracture extended down to the knee joint, separating the condyles of the femur. He therefore hesitated not to propose amputation. The surgeons present opposed the Baron's proposal, and the colonel was undecided. After a few moments reflexion, however, this officer peremptorily requested Baron Larrey to take off the limb. He did so, and dissection shewed the condyles separated, the knee joint filled with black blood, and the popliteal artery lacerated. Three other similar cases presented themselves on the same day.

Our excellent author preserved the knee joint in a great many cases, on this fatal day, where the leg was carried off, or disorganized so close to the knee that, without much previous experience, amputation of the thigh would have been resorted to. He had often before witnessed the good effects of amputating through the head of the tibia (dans l'épaisseur des condyles du tibia) and thus preserving the knee joint. In the present campaign such amputations had an immense superiority over those practised above the knee, on account of the greater facility with which the patients supplied themselves with wooden legs, and in consequence, preserved their lives (some of them at least) in the disastrous retreat.

During the first 24 hours our author amputated about *two hundred limbs*, which he thinks would have proved successful, in general, had the wounded even common accommodations. But, alas! they were totally destitute of every comfort and almost necessary of life; and far, far from any place that could afford them succour! Humanity will long bewail the miseries of these brave but unfortunate warriors, who deserved a better fate than to perish in the wilds of Russia, the victims of insatiate ambition in their chief! The Baron emphatically complains that the few resources in their power were not applied by the proper officer on this melancholy occasion.

Pursuing the enemy, the French army passed through Mozaisk, which they found, as usual, in flames; and in which, horrible to relate, were great numbers of wounded Russians perishing with hunger and thirst! Our author and a few humane soldiers administered to the wants and wounds of these wretches, thus deserted by their own medical and military officers, although their means were inadequate to the succour of their own countrymen.

Scarcely were the French army advanced a few miles from Mozaisk when they were astonished to find themselves (though in the immediate vicinity of one of the great capitals of the world) traversing sandy plains, arid and desert as those of Arabia. The mournful aspect of this solitude cast a gloom of despondency over the whole army, and seemed to prognosticate that Moscow itself, whose riches hitherto inspired them with hopes, would be found in ruins. During the march over this desert, men and horses sustained the most dreadful privations and fatigues, and thousands (particularly of the young conscripts) fell on the road. At length they reached Moscow on the 14th September, but found that the Russian army had drawn with them the almost entire population, and the French paraded the principal streets without meeting a human being! But what was more appaling, fires were seen to rise in various quarters where the French soldiers had not even penetrated, and particularly in the bazar of the Kremlin. The invading army, however, had just time to see and admire the noble buildings, elegant streets, and ample magazines of all kinds, with which this metropolis abounded, before the flames converted them into ruins. We dare not enter on Baron Larry's description of this dreadful and magnificent conflagration, lest we should be drawn too far from the more direct object of our labours—the collection of scientific information.

It may be permitted us to remark one or two circumstances. It will hardly be credited that the Russian incendiaries continued, in the face of the French, (who hung them up or shot them in all directions,) to set fire to the houses till the whole city was one universal blaze. "La peine de mort, appliquée à ceux qu'on prenait en flagrant délit, ne faisait nulle impression sur les autres; et l'incendie continua trois jours et trois nuits sans interruption." 73. Although Moscow was nearly reduced to ashes in eight days, yet when the fire ceased, and the French came to examine the ruins, abundance of every kind of provisions, furs, clothing, &c. were found in cellars and other subterraneous magazines—sufficient, Baron Larrey asserts, to supply the whole army till the ensuing summer! Thus it is evident that Napoleon's timidity in abandoning Moscow, was equal to his temerity in approaching it! Another fatality attending the French councils was, that even while they did remain in Moscow the magazines were hoarded, and the soldiery half starved —which magazines they burnt or left behind them at last! "Quem deus vult perdere prius dementat." In abandoning the city, however, every cart, carriage, knapsack, and even pocket, was crammed with Muscovite spoils. Darius's army, on departing from Babylon, presented not such a quantity of riches. But the weather and the roads soon shewed them that they must part with all their pillage, if they meant to regain their native soil. The army, however, presently got into a fine cultivated and inhabited country on the southern or Ukraine road; but here again Napoleon's evil star crossed him, and he determined to stretch northward, and return by the desert through which he advanced upon Moscow—"un grand sujet de chagrin pour toute l'armée." Here the tragic scene opens in earnest, and the horrors of this retreat baffle all human description! It was the cold that was their principal enemy; then famine, fatigue, the sword, &c. The Baron remarks that he attributes his own preservation entirely to the salutary habit which he had of marching on foot, in general, rather than on horseback. Those, on the other hand, who observed not this precaution, were so benumbed with the cold, that when the bivouac fires were lighted, they did not feel the heat till the seeds of gangrene were sown. The Baron walked almost the whole of the way, and never approached a fire.*

Of all the dreadful scenes that occurred during this memorable retreat, the passage of the Berezina was the most terrific. The bridges broken down, and the enemy pressing

---

* In a recent work published by Surgeon Begin, who accompanied this disastrous expedition, the following incident arrested our attention, and is not misplaced here.

"After leaving Moscow," says M. Begin, "we found all the villages in ashes, and a dead silence reigning every where around us. Having wandered a little from the main route of the army, I was roused from a melancholy reverie on the misfortunes of our army, by the groans of a human being who appeared, by the sounds, to be close to me. I stared around, but could see nothing, except scattered and half-putrified corpses. The groans continued, and I alighted from my horse to make a more careful examination of the place. After several minutes' search, I discovered in the ditch of a redoubt, and lodged in the disembowelled carcase of a horse, a wretched Russian soldier, whose right leg had been carried off by a cannon shot, and who had existed in that horrid asylum for six weeks—namely, from the battle of Moscowa! During this time he had lived on the carcase of the animal, whose bones and skin served him for a habitation. Almost every particle of flesh was clean scraped from the interior of the animal, the thorax and abdomen of which protected the wounded soldier from the pelting storm. The stump was nearly healed by the efforts of Nature alone, and the Russian, though pale, squalid, and haggard, was apparently firm in strength, and by no means ill in health." All M. Begin could do, was to give him a dram from his canteen, which set the poor Muscovite almost in ecstacies. He left him where he found him, but had no doubt that the Russian army who were pursuing them, would relieve the unfortunate soldier from his dreary abode in a day or two afterwards.

in all directions on the flanks and rear, caused the most dreadful havoc and confusion—nothing was to be heard but the most lamentable cries of thousands trodden under foot by their stronger neighbours—nothing to be seen but desperation and despair! On this fatal day, Baron Larrey nearly fell a sacrifice to his anxiety to preserve some cases of surgical instruments. The professional respect in which he was held saved his life. No sooner was he recognized than the French soldiers, regardless of their own safety, passed him along over their own heads, from one to another, till he crossed the only crazy bridge remaining, and which was completely blockaded up with military materials, and the bodies of the living and the dead.

"J'étais près de périr dans la foule à mon tour, lorsqu'heureusement je fus reconnu; ausitôt chacun s'empresse de favoriser mes efforts; transporté par les soldats de l'un à l'autre, je me trouvai, à ma grande surprise, en peu de momens sur le pont. Ce témoignage, qu'ils me donnèrent de leur attachement dans cette circonstance, me fit bientôt oublier et les dangers que j'avais courus et la perte que je venais de faire de mes équipages. 101.

The passage of the Berizina seems to have completed the panic of Napoleon, for he immediately afterwards abandoned his army, and made the best of his way back to France! The Russians pursued the remains of this ill-fated army no farther than Wilna. And now out of 400,000 fighting men, about *three thousand* of the guards remained!

"Trois mille hommes des meilleurs soldats de la garde, tant d'infanterie que de cavalerie, presque tous des contrées méridionales de la France étaient les seuls qui eussent vraiment résisté aux cruelles vicissitudes de la retraite; ils possédaient encore leurs armes, leurs chevaux et leur attitude guerrière: les maréchaux ducs de Dantzick et d'Istrie étaient à leur tête; les princes Joachim et Eugène marchaient au centre de cette troupe, que l'on pouvait considérer comme le reste d'une armée de plus de 400,000 hommes, que les habitans du pais avaient vue défiler, six mois auparavant, dans toute sa force et dans tout son éclat. L'honneur et la gloire des armées françaises s'étaient en quelque sorte retranchés dans ce petit corps d'élite." 114.

Our analytical course shall now be more strictly professional. In our author's circular to the French surgeons, speaking of the effects of intense cold, he observes, that, in general, frost-bitten sores (plais de congelation) present the same phenomena as burns. In both cases a gangrenous eschar, more or less extensive, is formed, the separation of which must be promoted by topical applications which excite the surrounding sound parts. The most simple and effectual dressing was unguentum de styrace, spread on linen or lint. Alcoholic embrocations and decoctions of cinchona counteracted the process employed by nature in the cure. The eschars separated, the wounds are to be considered as simple, and treated accordingly. Where amputation is necessary, from the irreparable destruction of a member, Baron Larrey does not advise the attempt at quickly uniting the integuments covering the stump; but his reasons for this do not appear very clear to us. Our author observed that the inhabitants of the more southern parts of France and of Europe bore the intense cold of Russia better than those born in the northern and colder latitudes. It is a curious but certain fact, that when our soldiers or sailors return from our tropical colonies, they feel the first winter's cold in England much less than the subsequent ones.

Of the very few who withstood the rigours of this fatal winter, a considerable proportion were destined, on reaching a friendly soil and having a supply of clothes and provisions, to encounter a new malady, which the medical officers denominated "*fievre meningite catarrhale de congelation.*" This disease, in a short time, became epidemic—and ultimately contagious. Our author traces the cause, properly enough, to the sudden change from cold and famine, to warmth and food—the result of which was an engorgement of the membranes, more especially of the mucous membrane of the lungs, and the meninges of the brain. These two principal effects were announced by tensive pains in the head with sense of heaviness;—lesion of the mental faculties and organs of sense; general debility; extreme anxiety. Cough now came on, and increased rapidly, accompanied by mucous, sometimes bloody expectoration. Occasional diarrhœa, inclination to vomit, and colicky pains supervened. The pulse was quick, the skin dry, the limbs benumbed, cramped, or spasmed. Sleep was laborious and disturbed with frightful dreams; the vessels of the eye were injected; fever became developed, with evening exacerbations, and great throbbing of the carotid and temporal arteries; delirium or lethargy ensued, and the danger was then imminent. When the issue was favourable, the inflammatory stage was generally of short duration, and was commonly terminated by a nasal hæmorrhage or diarrhœa, occurring on the fifth or ninth day. Sometimes, instead of these critical discharges from the mucous membranes, there was an abundant perspiration of a brownish colour, which tinged the patient's linen. When, on the other hand, the issue was fatal, apoplectic symptoms appeared, and proceeded with great rapidity, while erysipelatous spots shewed themselves on the lower extremities, and soon became gangrenous. The urine was very scanty, and of a blackish tint

—the alvine evacuations fetid and black—all the natural functions became interrupted, and the patient sunk on or before the fifth day.

Our author had many opportunities of examining the bodies of those who died of this disease. On the surface of the brain were found layers of albuminous substance, but without any points of suppuration—the sinuses of the dura mater full of coagulable blood—the brain firmer than natural, and its vessels injected—the mucous membrane of the larynx and bronchia of a dark brown colour in some places—the intestines very much contracted—and gangrenous spots, very generally, on the lower extremities and abdominal parietes. Baron Larrey experienced a severe attack of this fever himself, after visiting several hospitals in which great numbers were confined. After suffering dreadfully from excruciating head-aches, with occasional delirium, begging, in vain, to have the jugular vein opened, he was relieved by a copious and spontaneous hæmorrhage from the nose. He considers that the best mode of treatment was local and general blood-letting—cold applications to the head—warm fomentations and sinapisms to the feet—and, after the febrile excitement was subdued, some gastric derangement remaining, the administration of a gentle vomit of ipecacuan, sharpened with a small proportion of tartrite of antimony. After this, gentle tonics and nourishing diet completed the cure. The most trifling excess during convalescence, was sure to produce a relapse. Those who recovered from this disease, generally lost their hair; and in one case, that of a surgeon, the nails of the fingers and toes dropt off. The disease spread, after some time, to the inhabitants of those towns where the French soldiers were confined, thus proving the contagious property which became superadded to its original character.

In describing the cases of some wounded men in the hospitals of Dresden, the Baron informs us that the practice of the Saxon surgeons, in amputations, was, to cut the integuments and muscles at one incision, down to the bone, the latter being sawn off very little above the level of the soft parts. The surfaces of the stumps were then brought into close contact, and thus retained by sutures and bandages. The tourniquet was then, and not till then, slacked off. No arteries were taken up, and no hæmorrhage, in general, ensued.

We are now to take leave of military movements, and endeavour to convey to the English reader some of the practical results of our able author's experience—an experience of thirty years service, and twenty-four campaigns!

1. *Reflections on Wounds of the Head, and their consequences.*—Our author proposes to investigate, 1st. Those cases demanding the trephine. 2d. Those cases where it is useless or detrimental, though formerly or still recommended by authors. 3d. Hernia cerebri. 4th. Abscess in the liver supervening on wounds of the head.

Baron Larrey thinks the trephine indispensible where, in fractures of the cranium, the fragments are displaced, and driven in so as to injure the dura mater and brain. Also, where the foreign body which has caused the wound is entangled in the fracture, or has penetrated, but to no great depth, into the encephalon. Finally, where we are sure that extravasation exists beneath the cranium. One of the most common symptoms of compression is paralysis of parts on the opposite side; and this symptom is the more unequivocal in proportion as it declares itself immediately after the accident, and gradually increases in degree. We shall pass over the cases which Baron Larrey relates to show the the necessity of applying the trephine to remove foreign bodies and give vent to extravasated fluids, because the indications were sufficiently evident. We proceed to his *second* proposition. We should *not* apply the trephine, he avers, in cases of wounds of the head with fracture of the cranium, however extensive or stellated that fracture may be—provided the fragments are not driven in upon the soft parts;—no foreign bodies lodged;—and no symptoms of compression very evident.

The cerebral commotion, he justly remarks, is comparatively much less in cases of large wounds of the head with loss of substance in the soft parts, and fracture of the bones, because (he thinks) the effects of the percussion resulting from the impression of the wounding body are lost or expended, as it were, on the more exterior wounded parts, particularly if the said body has struck the cranial dome in a diagonal direction. In these cases the central mass of the brain being spared, effused fluids are more readily absorbed—the fractured pieces afterwards approach each other—and the accident is cured by the sole efforts of Nature. Under such circumstances the application of the trephine would retard rather than accelerate the healing process. Among the bad effects of the trephine, our author considers the laceration of the perecranium by the saw or other instrument, as the most dangerous. This operation augments the irritation of the contiguous membranes, and often determines those sympathetic affections (abscess in the liver for example) which supervene on wounds of the head. Here the Baron relates a very interesting case illustrative of the propriety of the non-trephining practice, of which we shall lay a brief abstract before our readers.

"In May 1812, near Berlin, a store-keeper was run against by a carriage going very rapidly, and had his head dashed against a sharp stone, which stripped the skull of integuments, and in some places, of pericranium, from the forehead to the tuberosity of the occiput. Immense flaps of integuments hung down over the neck and the ears. There was found a stellated fracture of the left frontal bone, one line of which was continued into the parietal bone of the same side. There did not appear any depression or displacement of bone. The man had a great hæmorrhage from the nose and ears, and preserved his senses. He was dressed in a clumsy manner, and great masses of charpee had been put between the flaps and cranium. The following day, being very ill and delirious, with acute pain, hard quick pulse, and flushed countenance, Baron Larrey was sent for. He removed all the dressings, and freely scarified the inflamed integuments. Common emollient dressings were applied, the patient was bled from the foot, and laxative drinks and lavements exhibited. The attendant surgeons had the trephining apparatus all ready when the Baron arrived; but the operation was deferred. The means abovementioned removed the pain and delirium, and the patient remained tranquil till night, when new symptoms of inflammation arose and rapidly increased. He was now bled from the jugular vein, and had mucilaginous, sedative, and antispasmodic drinks prescribed, by which he was relieved, and passed the night quietly. During the two succeeding days there were strong febrile movements in the system; but a sero-purulent discharge was soon established in the wounds, and became abundant, accompanied with much debility. A low fever was now ushered in with shiverings, pains, and nausea; succeeded by intense heat, thirst, and head-ache. Ice cold applications were made to the head and surface of the body, with cold drinks, &c. The alarming symptoms subsided, and were succeeded by some bilious vomitings and involuntary stools. This moment was seized for the exhibition of an emetic, which evacuated the stomach and bowels of great quantities of fetid matters. In the following night there was another severe exacerbation, with fixed pains in the forehead, and delirium. Cupping glasses with scarificators relieved these symptoms greatly, and ice-cold applications were kept to the head. Next day the pyrexial symptoms had entirely subsided, but such alarming debility succeeded, as gave our author reason to apprehend typhoid fever. A large blister to the nape of the neck; and bark with infusion of arnica montana internally. Some wine was also allowed. Next day, (the 26th of the accident,) the patient appeared perfectly sensible, and the wound was filling with healthy granulations. From this time he slowly, but progressively, recovered." 197.

Baron Larrey remarks that if, according to the direction of authors, and the advice of several surgeons who had seen the case, the trephine had here been applied, there is little doubt but that the operation would have exasperated the inflammation of the dura mater, (which certainly existed,) and thus proved destructive of life. From this and numerous other cases which have fallen under his notice, the Baron concludes that the operation of trephining is but seldom necessary, and that it should never be put in force if there be reason to apprehend inflammation of the cerebral membranes. The Baron therefore inquires, in this place, what is the proper period for the operation, in those cases where it is proper or necessary? Inflammation, he thinks, of the membranes of the brain generally establishes itself after twenty-four hours from the injury; consequently the operation should be practised as early as possible in that period. If inflammation have commenced, the operation must be delayed till the symptoms of it are removed; for even the presence of foreign bodies compressing the cerebral mass is less dangerous than the attemps to remove them; and the patient has less chance of recovery, in the latter case, than if he were left entirely to the resources of Nature. When the operation is performed, the dressings should be extremely simple, the bowels kept open, the functions of the skin and the internal secreting organs attended to, and the most aqueous regimen enjoined.

*Hernia Cerebri.* What is the cause of this phenomenon? Without pretending to ascertain this exactly, Baron Larrey hopes to point out the road towards truth. He considers this protrusion as owing to an inordinate excitement of the vessels of the membranes and brain in the vicinity of the wound, caused either by the irritation of foreign bodies, or the access of atmospheric air. He has never seen recoveries from large herniary protrusions of this kind; and the means taken to cure the complaint, such as compression, excision, caustic, &c. always aggravated or accelerated the evil. The Baron therefore advises surgeons to defend the hernia from the external air by pieces of fine linen soaked in camphorated oil; to remove all foreign and irritating bodies; and to lessen general and local irritation of the system, and of the brain, by diluent drinks, laxatives, quietude, &c. By these means Nature will herself reduce the hernia, when it is reducible.

We may take this opportunity of stating that Mr. Astley Cooper has long treated Hernia Cerebri with considerable success—that is, with success in three cases out of four, in the following manner:—if the hernia protrude but little beyond the level of the integuments, a dossil of lint dipped in lime-water is applied, and over that the capular bandage, with a moderate degree of pressure. If, however, the hernia protrude farther, and overlap the surrounding integuments, a silk thread is passed round the tumour and drawn tight so as to cut the vessels, and, in fact, remove the protruding portion. After this the dressing and moderate pressure are applied as above described. Mr. Cooper has been so kind as to inform us that in *young* people the foregoing plan will be crowned with success in the great majority of cases; but in *old* people it will be very much otherwise—for in them, all accidents about the head are dangerous, and too often fatal.

Here the Baron digresses, for a moment, from Hernia Cerebri, to relate two cases of wounds of the brain, for which he could not find a better place.

"During the campaign of Moscow, a grenadier of the ex-guard received a wound in the head from a Cossac's lance, which entered near the junction of the posterior superior angle of the left parietal bone with the occiput. The weapon was so sharp that it pierced the skull without the least splintering, and penetrated deeply into the brain. The man was left for dead on the field, but carried a few hours afterwards into a neighbouring town, where he continued for a considerable time insensible. The lance was withdrawn, the wound dressed, and went on to cicatrization without accident. The intellectual faculties were not injured, but it was evident that the wound had destroyed the functions of certain nerves, as the glosso-pharyngeus, par vagum, hypoglossus, spinal and suboccipital. The voice, at first hoarse, failed altogether in the end. Deglutition was difficult—the sense of taste and smell became enfeebled—the muscles of the larynx became partially paralysed, so that the larynx itself subsided half an inch lower down in the neck, dragging, straightening, and distorting the epiglottis and glottis, so that the patient was obliged to make extraordinary efforts to breathe by calling in the aid of various unusual muscles. The diaphragm participating in the paralysis could not act in dilating the cavity of the thorax, and the patient could not breathe without firmly closing the jaws, so as to draw up and widen the rima glottidis.—In short, the man would have been suffocated had his jaws been kept open for any length of time. The pharynx, œsophagus, and stomach, participated also in the paralysis, for deglutition was difficult, and emetics administered in large doses had no operative effect on the stomach. The abdomen evinced no alternate rise and fall corresponding with the respiratory muscles, as in other people; and when he attempted the least exercise, his face became discoloured, his body covered with perspiration, his extremities cold, the action of the heart slow, and the pulse scarce perceptible. When he sits down in quiet he recovers, in a great degree, from this state, and breathes with more freedom. *His digestion is very slow and difficult—he is obliged to eat very little and often, and to make use only of the most easily digestible substances.* He is threatened with marasmus." P. 209.

The other wound (by the point of a foil) which penetrated through the cribriform plate of the ethmoid bone, and went five or six lines into the base of the brain, was accompanied by many curious phenomena, which we have not space to state here, such as double vision, loss of smell for a time, hemiplegia of the opposite side, total forgetfulness of noun substantives, &c. &c.

II. *Hepatic Abscess succeeding wounds of the head.* It is well known that the author of "*Nosographie Chirurgicale*" made some notable experiments, by hurling thirty or forty dead bodies from various heights upon the stone floors, so as to fracture their skulls and lacerate their livers by the concussions; and thus, as he very knowingly supposed, explaining the phenomena of abscess in the liver succeeding gun-shot wounds of the head! We need not wonder at the absurdity of this analogy, when we recollect the extravagance of a thousand others pressed into the service of medicine and surgery. Baron Larrey, besides pointing out many defects and incongruities in the above mechanical hypothesis, shews that falls *during life* are very different in their effects on the liver, from those which take place after life has ceased, and when, in fact, the structure of the liver loses its main cohesive powers. He relates several cases in proof of this, where men fell from great heights by accident or design, and where the cranium and sometimes the limbs were crushed to pieces, yet no lesion of the liver was observable on dissection. In short, it is only where the hypochondrium comes in contact with some hard body in the fall, that rupture of the liver is frequent, when the accident happens to the living subject. On the other hand, as the Baron observes, abscesses in this organ are often seen to follow slight wounds of the head, unaccompanied by fracture, and unattended by any violent commotion of the system.

For a long time past Baron Larrey has had occasion to observe that the respiratory and biliary organs, but particularly the latter, were disturbed in their functions, and decidedly influenced by inflammation of the fibrous membranes of the brain and extremities. It appeared to him that the irritation established in a portion of the above membranes, was rapidly propagated, sympathetically, to the centres of those organs supplied by the ganglionic nerves;—and the liver being the most complex apparatus, with a peculiar capillary circulation, and copiously furnished with nerves from the great intercostal, seems the most disposed to receive this sympathetic irritation. Inflammation is kindled up, and quickly terminates in suppuration, which generally kills the patient, if he escape the effects of the traumatic inflammation.

We shall here condense one or two of Baron Larrey's cases in illustration of the present subject.

"*Case.* A Prussian soldier, in the hospital of Gros Caillou, in the year 1814, had a false articulation in the middle of the right os humeri, in consequence of a fracture of that bone some months previously. With the hope of obtaining a reunion of the bones, a seton was passed through between their ends. Inflammation shewed itself before the fifth day, and rose rapidly, with great tumefaction of the whole member from the shoulder to the fingers. To these symptoms were added acute pains in the right hypochondrium, with difficulty of breathing, oppression, and violent traumatic fever. The seton was withdrawn, emollient fomentations applied to the arm, and cupping glasses to the side, with diluent drinks, &c. These means were unsuccessful—the symptoms became exasperated—gangrene took place in the arm—lancinating pain was complained of in the region of the liver. In a few days a tumour appeared under the false ribs, displaying an evident fluctuation and unequivocal marks of hepatic abscess. The state of debility to which the patient was reduced prevented the attempt of amputation of the arm or opening the abscess, and the patient died in 24 hours from this time. On dissection, an enormous suppuration was found occupying the right lobe of the liver, and ready to burst into the abdominal cavity. This abscess must have been occasioned by the inflammation of the arm, for the man was in perfect bodily health when the seton was introduced." 231.

The three following cases were successively received into the hospital of Gros Caillou, for sabre wounds inflicted in duelling. The first was a young chasseur, who had an oval piece of the external table and diploe of the right parietal bone removed by a sabre; the internal table remained entire. The wound was treated as a simple one, and the patient put on low diet and diluent drinks. The first ten days passed without accident, but on the eleventh, the suppuration in the wound ceased, and the edges became swelled and red. To these were added fever, head-ache, tingling of the ears, delirium, thirst, and deep-seated pain in the right hypochondrium. Leeches were applied round the wound—the right side and the temples were cupped; the head was covered with an emollient cataplasm, pediluvia and lavements were employed, and antimoniated drinks prescribed. In spite of these means, however, the inflammation advanced rapidly—the pains in the side became lancinating—shiverings took place, with cold sweats, hectic fever, &c. of which the patient died on the 31st day from the accident. On dissection the pericranium was found highly inflamed, as also the dura mater beneath the wound of the bone. The brain was not diseased. On opening the abdomen, a quantity of purulent matter was found extravasated, and which had come from an enormous abscess seated in the convex side of the liver, and penetrating deeply into its substance.

A few days after this, a dragoon of the guard was brought to the hospital, who had a pretty thick scale of bone cut from the os occipitis by a sabre, the inner table escaping. The wound appeared slight, and he was placed in one of the convalescent wards, having been dressed in a simple manner. The wound was in a very good state, and cicatrizing kindly, when, all at once, inflammation arose—the right hypochondrium became painful—fever was kindled up, and all these symptoms quickly increased in violence. At this time Baron Larrey was called in, and prescribed *local* bleeding, cooling laxatives, pediluvia, and *antispasmodics*, but without effect; (at which we do not wonder, for certainly more vigorous means would have been used by British practitioners on such an occasion.) Dissection shewed violent inflammation of the pericranium and opposite portion of dura mater—considerable abscess in the liver—effusion of purulent matter into the cavity of the abdomen. Thus a man in the prime of life died of phrenitis and hepatitis, under one of the ablest physicians in France; and a vein was never opened during the course of his disease! Would any man believe this, were it not recorded by Baron Larrey himself? We rarely declaim, in general terms, against our neighbours on the Continent—we simply prove their maltreatment of diseases by their own symptomatology and dissections. Let them exonerate themselves from the charge if they can.

The last case we shall notice of this kind, was a grenadier, who was brought to the Gros Caillou hospital, with a sabre wound of the os frontis penetrating only as far as the diploe, and accompanied, for a short time, at first, with slight symptoms of commotion. The first ten days passed without accident; but at this period he complained of acute and continued pain in the wound, the suppuration being all at once suppressed therein. He had somnolency interrupted by convulsive movements and slight tendency to delirium; at the same time the patient complained of oppression, and dull constant pain in the right hypochondrium. Local blood-letting—diluents—laxatives—emollients. But symptoms of compression of the brain came on, with paralysis of the left *arm*, and continual agitation of the left *leg*. The right hypochondrium soon bulged out, while rigors and inclination to vomit shewed deep-seated mischief in the liver. The trephine was applied over the most depending angle of the cranial wound, and issue was thereby given to a spoonful of purulent matter mixed with blood, which was lodged betwixt the bone and dura mater. It is needless to observe that the hepatic affection soon terminated the patient's existence; and on dissection, effusion of purulent matter was found in the abdomen, from a large abscess in the substance of the liver.

It may be remarked that none of these wounded men experienced any fall, or other shock to the hepatic system, which could at all account for the abscess in the liver.

III. Among the wounds of the *throat*, there were some presenting curious phenomena. We shall abridge one or two, as specimens.

"An infantry officer presented himself with a gun-shot wound situated in the left side of the larynx, and taking a direction downwards and inwards, passing under the thyroid cartilage, which appeared slightly injured. The track of the ball appeared to descend under the trachea towards the chest, where the projectile was lost. The patient was harrassed with a constant oppressive pain, and difficulty of respiration, accompanied with redness of the face, turgescence of the neck, impossibility of swallowing solid food, and great difficulty in swallowing liquids. The probe gave no indication of the seat of the ball, but the patient always pointed to the site of the cricoid cartilage as the point of suffering. Nothwithstanding the vicinity of the thyroid arteries, Baron Larrey enlarged the wound upwards and downwards, without however procuring any advantage. He therefore concluded that the ball was lodged behind the bifurcation of the trachea, and not in any part of the cavity of the larynx. A few days afterwards the ball presented itself at the bottom of the wound, and was fortunately seized with the forceps and extracted. From this moment the patient was relieved, and went out of the hospital cured." 247.

Another case was that of a young sharpshooter, who was wounded in the neck. His state of suffocation indicated the imminent danger in which the patient was, when he presented himself to the surgeon. Speech was abolished, and he appeared in the anguish of death. Respiration was almost extinct, and at each effort at expiration, some air-bubbles, mixed with frothy blood, exuded from a gun-shot wound in the neck, on the left side of the larynx, between the os hyoides and thyroid cartilage, the os hyoides being fractured. The bullet had traversed the throat, and issued behind the angle of the lower jaw on the opposite side. The patient had lost much blood, and suffocation seemed to be threatened by the fluid accumulating in the larynx, the rima glottidis being wounded. The surgeon (M. Emangard) was much embarrassed; but luckily thought of making an opening anteriorly into the thyroid cartilage. The moment this was done, air and clots of blood rushed out. The swelling and engorgement of the neighbouring parts quickly subsided—respiration was rendered comparatively free, and the patient was snatched from the jaws of death. The wound was cleared by degrees, and the functions of the parts gradually restored, with the exception of the voice; and the patient conducted to a cure in a short time. 248.

IV. *Wounds of the Chest.* If, says Baron Larrey, a few people escape the fatal consequences of balls lodging in the cavity of the chest, they are, nevertheless, greatly harrassed by the irritation of the foreign body occasioning a source of purulent secretion, and too often empyema. If the passage by which the ball entered remain open or fistulous, the matter is discharged with more or less facility, according to the position, and the prognosis is thereby rendered more or less doubtful. In all cases, Nature endeavours to expel the foreign body, or prevent it from irritating the neighbouring parts—in the first case, by establishing suppuration round it, the ball falling to the lowest point of the abscess, and there causing fresh suppuration till it finds its way to the surface or into the hollow of some organ. This process is too often accompanied by hectic fever, emaciation, or even death. When the ball continues loose in the cavity of the chest, it will cause the death of the patient, if he be not relieved by art. At an early period the ball may be extracted between the ribs; but after some time, the ribs get contracted in their intervals, so as not to leave room for the extraction. In this case, a portion of rib itself must be removed—but neither the saw nor trephine is the instrument for this purpose. The lenticular knife used in trephining the skull, is the most proper, and there is much less difficulty in detaching a part of rib, (unless the patient be very old,) than might, *a priori*, be imagined.

*Case.* " A young voltigeur of the ex-guard received, in one of the battles near Paris, a musket shot, which cut away the upper half of the fourth rib, at about an inch from its sternal cartilage. The ball penetrated into the chest—traversed a portion of lung—and struck, without doubt, the spinal column, about the eighth or ninth vertebra, where its progress was arrested. This wound was accompanied with hæmorrhage, extravasation of blood, frequent faintings, oppression, anxiety, and spitting of blood—in fine, the patient said he was certainly going to die. He was transported to the Ambulances, in the vicinity of Paris, where he remained till the month of August of the same year, at which time he was sent to Gros Caillou, and came under the care of our author. At this epoch the patient presented a fistulous wound at the upper part of the right side of the chest, with evident empyema of that cavity. He was greatly exhausted and worn down by the hectic fever. The wound discharged, at each dressing, a considerable quantity of pus, which the patient himself expelled by a particular position of the body. His emaciation was extreme, and the fever exacerbated in the evenings. Baron Larrey introduced a flexible sound into the wound which went to the bottom of that side of the chest, without any resistance, and there struck against a hard body, which the Baron judged to be the ball. From its position our author judged it practicable to open into the thorax opposite the foreign body, and between the eighth and ninth ribs, counting from above. The operation for empyema being performed at the abovementioned place, about twelve ounces of pus came away, and the Baron discovered the ball, which was easily extracted by a pair of polypus-forceps, the intercostal space being ample and the ball rather flattened. The operation was followed by some formidable symptoms, but they were subdued in three days. The purulent discharge was considerable, but only issued from the inferior wound, the superior soon healing. Bark, in various forms, was administered, and the purulent secretion diminished daily, with evident restoration of the vital and natural functions—in short, this young soldier was on the point of being discharged cured, when, having committed a debauch in brandy, he was seized with enteritis, and died, one hundred days from the operation, and six months from the receipt of the wound. The dissection confirmed the suspicions of Baron Larrey relative to the first track of the ball." 258.

The *second case* of this kind was that of a young corporal, 26 years of age, who was wounded by a musket ball, at the battle of Moillow, in Russia, on the 22d July, 1812, the ball entering the chest between the eighth and ninth ribs of the right side, and lodging in the corresponding cavity. This soldier fell insensible on the spot, and remained two days on the field of battle, menaced all the time with suffocation:—at length he was carried to one of the hospitals of Moillow, three days after which he was on the point of perishing from the effects of an enormous effusion in the chest, but was providentially relieved by one of the French surgeons, who enlarged the original wound with a probe-pointed bistoury, and thus performed the operation of empyema which snatched the patient from the jaws of death. He was subsequently removed to the hospitals of Cowno, Koningsberg, and Thorn. A few weeks after arriving at the latter place, the wound having closed, for a short time, the patient experienced a renewal of the symptoms of compression and difficulty of breathing, which were relieved by a spontaneous discharge of matter from an abscess formed under the edges of the false ribs. From this orifice fragments of clothing were discharged with the purulent matter. But this channel soon closed, and one opened out again and continued discharging. The ball could not be found, and the patient dragged on a wretched existence in various hospitals, for the space of four years, (having been discharged incurable by a board of health,) when he was, at the intercession of Baron Larrey, admitted into the hospital of Gros Caillou, on the 15th June, 1816. On the first examination, Baron Larrey discovered the ball, by means of a sound introduced to the bottom of the right thoracic cavity. The purulent discharge was still copious from the fistulous wound, and the patient was suffering from incipient hectic fever. After having enlarged the wound, Baron Larrey made several vain attempts to extract the foreign body. The intercostal space was too confined, and the two ribs would not admit of the least separation. The Baron had moved the ball several times with the sound, and brought it near the wound, but it always slipped, and fell back into the cavity of the chest. How then was he to proceed? The anchylosed state of the ribs would not permit the application of a saw, (though made for the purpose,) without endangering the intercostal artery of the rib above, and the trephine, on trial, was found inadequate to remove a portion of rib, without which the forceps could not be introduced, nor the ball extracted. In this dilemma the Baron had recourse to the lenticular used in paring the bone after trephining. Having exposed about an inch and a half of the rib inferior to the wound, and tied a couple of arterial branches that bled freely, he introduced the lenticular knife, (the button being screwed off,) and sliced layer after layer from the superior edge of the said inferior rib, until he had formed a semi-lunar notch in it, five or six lines in depth. He now made fresh efforts to extract the ball, but the opening was still too small, as the Russian bullets weigh an ounce and a half, which is nearly double that of the French. He was therefore obliged to cut to within two lines of the inferior edge of the rib, not daring to go farther lest the intercostal artery should be wounded. A polypus forceps being now introduced, the ball was seized, and fortunately extracted, though with considerable difficulty, augmented by the ragged state of the ball itself. A warm aromatic injection was thrown into the cavity whence the ball was extracted, and the wound dressed superficially. In the night the patient (who supported the operation with great courage) suffered some distressing symptoms; and at the morning visit, Baron Larrey found him with fever, thirst, and general increase of temperature. He experienced acute and throbbing pain in the region of the liver, and throughout the whole track of the bullet. Blood was taken by cupping from the neighbouring parts, and the patient confined to acidulated drinks, &c. by which means the inflammatory symptoms were reduced, and the patient's sufferings relieved. A hæmorrhage took place from one of the arteries that had been tied, and this contributed to reduce the inflammation completely; but he appeared so weak, that slight tonics were prescribed. He continued two or three days in a very favourable progress towards recovery, when, happening to go by himself to the water-closet, he fractured, by a sudden incurvation of the body, the remaining portion of the ninth rib, and ruptured the intercostal artery, the hæmorrhage from which brought this unfortunate patient once more to the brink of the grave! Baron Larrey was immediately on the spot, and both arrested the bleeding and provided against its occurrence—"à l'aide de la bourse compressive et tres-ingenieuse de Desault." The pulse, however, was gone, the extremities cold, and all the symptoms of death present. Cordials restored the circulation, and perfect quietude preserved it. For two or three days he manifested symptoms of putrid fever, and was in great danger. The dressings were cautiously removed, and a quantity of purulent matter issued from the wound, which exhibited the phenomena of incipient hospital gangrene. Baron Larrey immediately exhibited a vomit, notwithstanding the precarious and peculiar situation of the patient. The emetic, which operated well, and discharged copious evacuations upwards and downwards, gave a sudden turn to the dangerous state of the patient. From this moment he felt himself better, and went on rapidly to convalescence and recovery. He was afterwards presented to the "Societé de Medécine," on the 22d August, 1816.

V. *Gun-Shot Wounds of the Abdomen.* Baron Larrey's attention has been much directed towards wounds penetrating the confines of chest and abdomen. Those of the right hypochondrium are more or less dangerous according to their depth: but in all cases where the liver is wounded, however slightly, there quickly supervenes such a disturbance of the vital functions as threatens the patient with imminent danger. "Where bile becomes extravasated in the abdominal cavity the case is mortal."

"At first (in wounds of the liver) the surface of the body becomes of a yellow tint, the eye hollow, dull, and watery;—the cold spreads from the extremities towards the trunk—nausea, hiccup, and anxiety are developed—the urine is bloody and scanty—the alvine excretions are devoid of bile—the pulse is small and weak—the voice fails—respiration is short, and more or less laborious. These symptoms become rapidly aggravated, and the patient dies, unless relieved by art." 270.

The first thing to be done is to enlarge and clear the wound, extract with caution all extraneous bodies, and to apply to the wound one or two cupping glasses exhausted of air; cupping, with scarifications, the neighbouring parts. These operations are to be repeated whenever the local pain assumes any degree of intensity. When the objects are accomplished, the lips of the wound are to be brought into contact, and held so by proper dressings. The dry and moist cupping, Baron Larrey observes, have the property of discharging not only the blood extravasated in the sinuses of the wound, but of disgorging the weakened vessels of the organ, favouring the resorption of effused fluids, and thus preventing inflammation. General blood-letting, he thinks, is not so advantageous, as it weakens the system without much relieving the part. The interior of the wound must be carefully preserved from the contact of air—the diet must be most rigorous, and the bowels kept open.

Where wounds of the abdominal parietes are accompanied with protrusion of a portion of omentum, Baron Larrey has advised the surgeon not to return it, nor yet remove it by knife or ligature. Soon after the accident, he observes, the protruded portion swells, thickens, reddens, and becomes rugous. These symptoms increase till the third day, after which they become stationary till about the fifteenth, when the part begins to shrivel, and ultimately is reduced without any operation. When the protruded part gangrenes, authors have recommended it to be removed by the knife, after passing a ligature round its base; but the Baron protests against this as unsafe and unnecessary.

" *Case.* A young officer was carried to Gros Caillou in August 1815, apparently expiring, with a sword wound penetrating the abdomen, accompanied with hæmorrhage, protrusion of the omentum, and injury of the stomach. Baron Larrey was called up to him in the middle of the night, and found a wound, about an inch and a half in extent, at the inferior part of the right hypochondrium, two inches from the cartilage of the eighth rib—a considerable portion of epiploon being protruded and strangulated by an irregular wound through the abdominal muscles. The pulse was feeble, the extremities cold, the vomitings frequent, the anxiety extreme, with hiccup, loss of voice and power of articulation—in short, every appearance of approaching dissolution. It was not deemed adviseable, under these circumstances, to attempt any operation. The night was passed in great distress; nevertheless, in the morning, the pulse and animal heat were, in some degree, restored; but the local pain was very acute, with bloody vomiting and hiccup. Baron Larrey now enlarged the wound of the integuments, without touching the omentum, which was thus liberated from strangulation, and the vomiting, &c. ceased. This done, the Baron enveloped the exposed omentum (as large as an apple) in linen soaked in warm wine—placed the patient in a proper position—prescribed acidulated drinks, lavemens, and embrocations to the abdomen. The alarming symptoms now subsided gradually, and after the fifth day he appeared out of danger. After the first 24 hours, the protruded epiploon swelled to nearly the size of one's fist, being red, rugous, and evincing signs of sensibility. It remained in this state till about the 10th day, when it began to diminish in volume and density, which was accelerated by graduated pressure through the aid of a compress. The epiplocele returned by little and little, till the whole was drawn into the abdomen, the wound cicatrizing as the omentum retreated. The officer was perfectly recovered by the 45th day from the accident." 281.

Another very interesting case is detailed, but we deem the foregoing one sufficient for our purpose.

VI. *Wounds of the bladder.* It is well known that the ancients considered wounds of this organ as mortal; but modern surgery has proved the contrary. When the bladder is void of urine, it is very difficult to wound it by any kind of instrument. But soldiers and sailors in the heat of action, particularly if the engagement be prolonged, are very apt to have this receptacle so filled as to present a conspicuous mark for sword or bullet entering the pelvis. When the bladder is penetrated in any part of its parietes *covered with peritoneum,* it is usually mortal. The urine escapes into the cavity of the abdomen in such cases, and death generally takes place in forty-eight hours. When the wound, however, is situated in any part *not* covered by peritoneum, it is not necessarily fatal, unless the internal hæmorrhage is excessive. In this class of lesions the urine occasionally escapes by the external wound, especially if a catheter be not kept in the urethra, a very necessary precaution to prevent this, and the extravasation of urine with its necessary consequence, the formation of abscesses. Warm baths and fomentations, with enemata and local bleeding, must not be neglected. We shall here introduce a summary view of two cases which are not devoid of interest.

"A chasseur of the ex-garde was struck in the upper and outer part of the thigh by a cossack's lance, while in the act of a cavalry-charge, the point of the lance glancing upwards and inwards, passing the inguinal glands, under the crural arch, and penetrating into the bladder, below the reflection of the peritoneum. The urine immediately followed the direction of the wound, and appeared at its orifice in the thigh; a few hours after which, the water was passed by the urethra with a considerable quantity of blood. To this hæmorrhage a state of quietude succeeded, the urine not appearing any more by the wound, the edges of which were brought into contact by proper dressings. Suppuration and an abscess became established in the track of the wound, and, when opened, a quantity of pus and urine was discharged—the wound remaining fistulous for some considerable time. Nevertheless, by means of the elastic catheter in the urethra, and proper dressings, the patient was conducted to perfect recovery." 289.

The other case was that of a French soldier, who, being half drunk, at one of the bull-fights in Spain, entered into the arena to contend with the enraged animal, in imitation of the professed fighters. He soon felt the consequences; for the irritated bull impaled him with one of its horns, and tossed him to a considerable distance behind! One of the intrepid combatants immediately darted on the enfuriated animal and killed him on the spot. Baron Larrey leaped into the arena, and was the first to give succour to the wounded soldier, who was lying insensible on the ground. He was quickly conveyed to the hospital, attended by the Baron, who found a torn wound, an inch and a half in extent, at the upper part of the right buttock, and taking a direction towards the lower part of the groin of the same side. On farther examination, the Baron ascertained that the horn of the animal, which was sharp and much curved, after having lacerated the cellular substance, and glands of the groin, had passed under the crural arch, penetrated deep into the pelvis, and wounded the bladder, which was full of urine at the time. The bladder, however, had not been pierced throughout all it coats—the inner one remained whole, and formed a hernia, or membranous pouch under the crual arch, as large as a pullet's egg. There was considerable hæmorrhage at first from the wound—the man was cold—his pulse very small—his anxiety great, and there was suppression of urine. After taking some coffee, he became sick and vomited, which disembarrassed his stomach of the wine, and relieved all the symptoms except the frequent inclination to make water. After clearing and enlarging the wound, and introducing a gum catheter by the urethra, Baron Larrey easily, though gradually, reduced the cystocele by gentle pressure. Tranquillity was restored, and the patient did well. He kept the catheter in the bladder, however, until the wound was healed. 291.

In respect to *gun-shot* wounds of the bladder, our author observes, that a ball may either pass through the bladder, and make its exit by the integuments, or lodge in the bladder itself. In the former case the urine escapes by one or both orifices, according to their situation. There is a diminution or total suppression of urine by the urethra, with discharge of blood by that passage, more or less considerable. The patient experiences very acute pain in the direction of the wounds—frequent and painful efforts to make water—nausea—sometimes vomiting—extreme anxiety and inquietude—paleness of the face—and sometimes plaintive cries. Not unfrequently the rectum is wounded, and the urine is voided by the anus. If the wound of the bladder be situated where covered by peritoneum, the extravasation of urine into the abdomen soon brings on inflammation there, and rapid death—often with apparent metastasis to the brain, and urinous odour through every part of the body.

Although the urine passes, at first, by wounds of the bladder, it is rare that any extravasations into the cellular membrane take place in the early periods of the accident, because the parietes of the track of the ball swell and become engorged, thus presenting a barrier to the infiltration. But when the sloughs are detached, the urine flows afresh along the track of the ball, and *then* it may penetrate into the surrounding cellular tissues, and cause serious mischief. This mischief can only be prevented by assiduously keeping a gum-catheter in the passage. Sometimes the rupture of arterial branches causes an effusion of blood into the bladder, and great irritation, heat, and inflammation. This accident is recognized by retention of urine, small pulse, paleness of the countenance, and dryness of the wound. The blood very rarely forms a coagulum in the bladder, on account of its constant dilution by the urine; consequently it may generally be drawn off by the catheter. Emollient and anodyne injections, at these times, are beneficial. At all times, Baron Larrey thinks, the entrance and exit of the ball should be well *debrided,* (scarified,) taking care not to injure parts of importance. This scarification is a *local bleeding* of the greatest advantage in repressing inflammation, and facilitating the subsequent separation of the sloughs. The three or four first days of wounded bladder are generally very severe; and, provided the urine flows by the proper channel, during this period, no catheter should be introduced. But at the moment when the sloughs are about to separate, the suppuration being established, then the catheter is to be passed into the bladder to prevent infiltration of the urine along the wounds. We shall abridge a case in illustration.

"The Sieur Burnot was wounded at the battle of Hanau, on the 30th October, 1813, by a musket bullet, which traversed the scrotum, tore the spermatic cord of the right side, fractured the inferior ramus of the pubis, cut the urethra, entered the bladder, traversed that organ and the rectum, and came out at the upper part of the left buttock, about an inch from the anus. The discharge of urine and stercoracious matters, both by the wounds and by the anus, left no doubt as to the double lesion of these viscera. The patient was conveyed to the hospital of Mayence, where Baron Larrey directed the treatment. The small quantity of urine which escaped by the entrance of the ball through the scrotum, had been sufficient to affect with gangrene the cellular tissue of those parts. The extirpation of the testicle, rendered necessary by the destruction of the cord, and the deep scarifications practised, arrested the progress of the gangrene. The sloughs were detached, all the bad symptoms subsided, and the patient escaped the first danger. Baron Larrey now passed into the bladder a hollow elastic catheter; prescribed lavemens; and ordered a proper regimen. The urine and stercoraceous matters had passed, for some time, by the posterior wound; and sometimes also by the urethra were passed small fragments of bone, accompanied by acute pain and some discharge of blood. The anterior wound healed first; the posterior continued a considerable time fistulous—the patient being long harrassed by "un flux diarrhêique urineux." The catheter was continued without intermission—the wounds cicatrized, and the patient got perfectly well." 298.

Baron Larrey quotes an interesting case related in the Memoirs of the Academy of Sciences, for the year 1725, proving that gun-shot wounds of the bladder are sometimes attended with dangerous hæmorrhages.

"A mason at Lausanne, 25 years of age, received a musket shot in the abdomen. The bullet entered about an inch from the pubis on the left side, and two fingers' breadth from the linea alba, penetrating the rectus muscle, epigastric artery, fundus of the bladder, and os sacrum, coming out near the anus. The spermatic cord was also wounded, but none of the intestines. The urine issued from both wounds. There was great hæmorrhage during the first few days; and there appeared to be an accumulation of blood in the bladder, producing pervigilium, delirium, ardent thirst, retention of urine, tension of the abdomen, and other dangerous symptoms. M. Martin, the surgeon, after trying a number of remedies in vain, had recourse to emollient injections into the bladder, which facilitated the expulsion of blood and urine, after which the dangerous symptoms quickly subsided."

When a ball lodges in the bladder, nothing, of course, but lithotomy can be depended on. Baron Larrey prefers the lateral; or, as he terms it, the *sub-pubic* operation, performed with the knife alone, and not the gorget,

VII. *Wounded Arteries.* The subject of wounded arteries has been studied with more attention in England than in France, and therefore we shall not dwell on any of Baron Larrey's didactic precepts. We shall merely introduce a few cases, abridged from the original.

"A young soldier of the ex-garde received a gun-shot wound in the left thigh, at the battle of Dresden, in 1813. The ball had traversed the lower third of the member from within outwards, destroying the femoral artery a few lines above where it passes through the triceps muscle. A considerable hæmorrhage took place at the moment of the injury, to which succeeded faintness, and abolition of pulsation in the popliteal artery. After scarrifying (débridé) the anterior wound, and removing some clots of blood that occupied the space between the two wounds, our author introduced his finger, and a void, where the femoral artery before ran, was now felt. Above the void, the torn and contracted extremity of the vessel was felt pulsating. As there was no appearance of new hæmorrhage, Baron Larrey contented himself with a simple dressing, and resolved to wait the event. No hæmorrhage occurred, and the wounds were both healed by the 61st day from the accident. The circulation in the extremity was gradually re-established, but no pulsation returned in the popliteal artery." 323.

The following case presents some interesting particulars.

"Daniel Hyppolite, a sergeant in the royal garde, received the thrust of a sword in the upper part of the right thigh, on the 9th of April, 1817, the weapon entering about three inches and a half below the anterior inferior spinous process of the ilium, and penetrating two or three inches in a horizontal direction inwardly, in the site of the femoral artery. The blood issued *per saltum*, in great quantity, and syncope put an end to the flow. Next day the hæmorrhage returned, with the same phenomena, and was restrained with the greatest difficulty. The patient was now sent to the hospital of the garde, and Baron Larrey visited him, when the following circumstances were noted:—viz. a tumour in the right groin the size of one's fist, extending from the anterior inferior spine of the ilium to the pubis, of a bluish colour, and pulsating synchronously with the other arteries. The patient experienced a sense of coldness in the leg and foot of that side, and a sensation of much heat in the tumour itself. A febrile movement was evident in the sanguiferous system. He was bled from the arm, and the operation repeated twice within the first twenty-four hours. He had cold drink for beverage, with a cold cataplasm to the tumour, while the limb was wrapped in hot flannel. Perfect rest and the horizontal position were enjoined. In two or three days the symptoms were mitigated, and the tumour began to diminish in size, the pulsations concentrating, and growing more feeble. Ice, inclosed in a bladder, was substituted for the cataplasm. The reduction of the tumour was succeeded by an acute pain in the knee and leg of the same side. The pulsations of the femoral artery in the thigh and at the ham had become greatly enfeebled, while the articular artery on the inside of the knee joint was felt beating very strongly. These considerations led to the belief that the crural artery was obliterated in a great part of its extent, and that a new line of circulation was established by anastomosing vessels. Nevertheless, the pulsation gradually returned in the femoral artery, while the increased size and action of the articular branches progressively decreased, evincing a restoration of the original channel of the circulation." 326.

We shall conclude our analysis with a pretty full detail of a very remarkable case of wounded artery.

Peter Cadrieux, 32 years of age, was wounded in a duel on the 20th November, 1811. The point of his adversary's sabre entered above the sterno-clavicular articulation of the left side, passed backwards and rather downwards, cutting a portion of the sterno-mastoideus and scalenus anticus muscles, wounding the subclavian artery, the subclavian vein, and probably a considerable portion of the brachial plexus of nerves. A tremendous hæmorrhage ensued, and syncope was the result. Being carried to a neighbouring house apparently lifeless, he was restored by wine and cordials, and then transported to the hospital of Gros Caillou. On the morning of the 21st Baron Larrey saw the patient, who presented all the symptoms of approaching dissolution. He was cold as marble—face pale as death—eyes expressionless—voice inarticulate. The wound, which was about three quarters of an inch in breadth, did not give out any blood; but a pulsating tumour, both above and below the clavicle, effaced all appearance of that bone. These pulsations, which were synchronous with the pulse, were more dictinct below than above the line which the clavicle was known to occupy. A thrilling sound was also distinctly heard in the direction of the axillary vein, which resembled that of a fluid passing through tortuous metallic tubes. The arm of that side was cold as ice, insensible, motionless, and without arterial pulsations. The pulse of the other arm was small, and hardly perceptible; yet the respiration was free, and gave no evidence of any extravasation in the chest. Although there was little expectation of life in this case, Baron Larrey hastened to have the arm chafed with warm chamomile oil, and then enveloped in hot flannels. The whole surface of the body was also chafed with hot and stimulating liniments, and nourishment was exhibited internally. By these means the powers of life and the natural temperature were gradually re-established. At the evening visit, Baron Larrey found the patient agitated and restless, with symptoms of approaching irritative fever. The tumour and the corresponding member were in the same state as before; but the jugular veins of that side were gorged and pulsating. Some demulcent and slightly anodyne drinks were prescribed for the night, and a surgeon was placed to observe what might happen.

22*d Nov. Morning.* The aneurismal tumor, without being augmented in size, was pulsating more strongly, as were the jugular of that side, and the carotids of both sides. The face was flushed, and the patient complained of a violent beating in the head. He was bled from the other arm —and compresses wet with gelid fluids were kept on the tumor,—emollient lavemens. The night between the 22d and the 23d was very restless.* At the morning visit of the 23d the vessels were found extremely gorged—the head-ache violent, and signs of approaching delirium—pulse of the sound arm febrile and nervous—(febrile et nerveux.) The left jugular vein was opened, and the blood flowed in an arch, of a vermillion colour, and with all the characters of arterial blood. This bleeding calmed all the symptoms, but still the patient could not sleep.

24*th.* All the symptoms were again greatly aggravated, and a third bleeding from the arm was practised. The insomnium and cephalalgia continued with trifling variation till the ninth day, one more venesection being performed in the interval. All this time the low regimen and external cold applications were persevered in, the state of the tumor and arm remaining in *statu quo.* The wound made by the sabre was cicatrized on the 8th day. On the tenth day the veins of the affected member began to smell—they had hitherto continued shrunk—the cephalic vein of this arm pulsated—the animal heat and sensibility became developed in the greater part of the member; but still there was no pulse. The aneurismal tumor was reduced in volume, and concentrated into a small space under the clavicle and behind the pectoral muscle; but the whizzing sound, before alluded to, was more audible than ever. Several days passed in this state, the pains of the head diminishing, the sleep returning, and the danger appearing to lessen. The hand still remained immoveable, and the patient experienced a

---

* The French have a curious but expressive term for a restless or bad night spent by a patient—they call it a *stormy* night. Thus, "la nuit du 22 au 23 fut *orageuse.*" When symptoms or diseases are *violent,* they term them *thundering*—"foudroyant." In some cases this term appears rather incongruous to the English ear. Thus, when an apoplectic patient is lying *speechless and motionless,* he is said by the French physician to have a—*thundering apoplexy.*

sense of most troublesome formication there, which no liniments or anodynes could assuage. The aneurismal tumor had entirely disappeared by the 20th day, the whizzing noise continuing the same, as also the pulsations in the veins of the arm and neck. The cold applications were now discontinued, and light nourishment was permitted. The heat, sensibility, and power of motion in the arm gradually returned, and about the 50th day slight pulsations were felt in the radial and ulnar arteries. The noise was diminishing, as was the pulsation of the brachial and jugular veins. This was the state of the patient when Baron Larrey presented him to the Society of the Faculty of Medicine, on the 56th day from the accident. On the Baron's return from Russia, in August 1815, he examined the patient, who, in the interval, had been placed in the hospital of invalids, and had been a second time presented to the society. The changes which had taken place in these four years were remarkable enough. In the *first* place the axillary, radial, and ulnar arteries, which, at the end of 56 days, had recovered, in a great measure, their pulsations, were now completely motionless, while the heat and nutrition of the limb had suffered no interruption—a fact which proves the formation of a new system of circulation in the member. In the *second* place, the pulsations, at first so remarkable in the brachial and jugular veins, have entirely ceased, and the vessels themselves, if still existing, are not visible. *Thirdly* the principal fingers of the hand are strongly retracted, and deprived of motion—probably, in Baron Larrey's opinion, owing to the wound of the brachial plexus. We shall not enter into the Baron's reflexions on the above curious case, as our surgical brethren will be able to turn it to account in their own minds. But we cannot help noticing a note which the Baron has inserted at page 353 of his work, which we shall literally translate. Speaking of the operation of tying the external iliac artery, first practised by Mr. Abernethy, and subsequently by Mr. Astley Cooper, and many others, the Baron observes :—

"This operation forms the subject of a question too important to be treated of here; but I will just say, by way of anticipation, that the dissections of *almost* all the subjects who had undergone this operation, shewed all the characters of an aneurismal diathesis—that is to say, there were found aneurismal tumours in other arteries at a distance from that on which the ligature had been placed. This being the case, I ask what purpose can the operation serve." 353.

We answer, in the first place, that Baron Larrey cannot possibly have seen one in ten of those who have had the external iliac artery taken up, and consequently his assertion is not founded on a proper basis. In the second place, if tying the external iliac artery be capable of *producing* what the Baron terms the aneurismal diathesis, it is quite evident that the ligature of the carotid inguinal, or other large artery, must be objectionable on the same principle. Yet we see all these arteries tied every year, and many of the patients living for a great many years afterwards, without the development of the aneurismal diathesis. If, on the other hand, he mean to infer that where an aneurism exists, requiring ligature of the external iliac, it may be inferred that there is probably also aneurismal dispositions in other arteries, then we say that experience does not universally, or, perhaps, generally support his inference, and that it is consequently untenable.

In respect to those cases which were operated on in this country, some certainly turned out to have an aneurismal diathesis, but many others not. Of the latter description we may mention two cases operated on by Mr. Brodie, one in the year 1813, the other in 1817. The former was seen five years after the operation, in good health—the latter three years after the artery was tied, and also in good health. These cases, were there no others, sanction the propriety, and demonstrate the *utility* of the operation, since one positive proof is worth a dozen of negative.

Baron Larrey must have been acquainted with the success which attended Mr. Astley Cooper's operations on the external iliac artery, as stated in M. Roux's Parallel, pages 275-6, and quoted in Cooper's Surgical Dictionary, 3d edition, p. 98. Since that statement appeared, Mr. Cooper twice performed this operation; once on a patient in Guy's Hospital, who died of secondary hæmorrhage, and again, two or three years ago, on Mr. Judd, a surgeon in Stamford, who is now living and following his profession in perfect health. In fact, there have now been nearly twenty successful cases of ligature of the external iliac artery by British surgeons, and therefore the propriety and utility of the operation are established on a broad basis of experience, which Baron Larry's evidence to the contrary can have no power to shake.

Baron Larrey's work concludes with a memoir on the advantages of the moxa and actual cautery in hip diseases and Rachialgia; but as these remedies do not seem likely to come into use, at least at present, in this country, we shall pass over the subject, more especially as the limits which we assigned for this article are exceeded.

We entertain a high respect for Baron Larrey's zeal, talents, and unbounded experience; and although we think that, on the subject of taking up the iliac artery, he has evinced something like a disposition to undervalue the intrepidity of British surgery, yet we attribute it to that *national pride* from which few of his, or perhaps of our own, countrymen are free. It is a pity, however, that such feelings should be able to insinuate themselves into the bosom of philosophy or science, and we hope the day is not far off, when they will be banished even from the breast of the politician. We must say, indeed, that, except in this one instance, Baron Larrey's volume is remarkably free from national prejudice of every kind, and abounds with interesting facts and useful observations, of which we trust we have given abundant proof in this analytical review.

